# Congenital melanocytic nevi in Bardet-Biedl syndrome

**DOI:** 10.1186/s13023-025-03870-6

**Published:** 2025-08-28

**Authors:** Karli Shelton, Phu Dang, Courtney McCorkle, Pooja Mallipaddi, Nicholas Hollman, Jeremy Pomeroy, Jesse Richards

**Affiliations:** 1 Department of Internal Medicine, OU-TU School of Community Medicine, Tulsa, USA; 2 OU-TU School of Community Medicine, Tulsa, USA; 3 Office for Research Development, OU-TU School of Community Medicine, Tulsa, USA; 4 Marshfield Clinic Health System, Marshfield, USA

**Keywords:** Obesity, Genetic disease, Bardet-Biedl, Congenital melanocytic nevi

## Abstract

**Background:**

Bardet-Biedl Syndrome (BBS) is a rare obesogenic disorder affecting multiple organs. The diagnosis of BBS is usually difficult and delayed due to this syndrome’s wide variety of clinical features. This study aims to assess the rate of congenital melanocytic nevi (CMN) in the BBS population in an effort to bring light to an easily assessable and early manifestation of BBS to aid in earlier diagnosis.

**Methods:**

We utilized a survey distributed to patients with BBS registered within the Clinical Registry Investigating Bardet-Biedl Syndrome database. Analysis was performed to identify participants with CMN and their prevalence of major and minor symptoms of the diagnostic criteria for BBS.

**Results:**

Data from 67 patients with BBS were gathered from our surveys. Of those participants, 23.9% reported having a CMN. Patients with CMN were more likely to have abnormal reproductive health issues, high arched palate, missing teeth, dental crowning, short teeth roots, and webbed fingers and toes.

**Conclusion:**

Our findings suggest that BBS is associated with CMN, possibly through altered neural crest cell migration. Screening for CMN shows a promise as a potential non-invasive screening tool to aid in earlier diagnosis of BBS.

**Supplementary Information:**

The online version contains supplementary material available at 10.1186/s13023-025-03870-6.

## Introduction

Bardet-Biedl Syndrome (BBS) is a rare genetic disorder, with an estimated prevalence ranging from 1:100,000 to 1:140,000 in North America and a higher incidence observed in males compared to females [1]. The syndrome is primarily characterized by a range of clinical manifestations, including obesity, retinopathy, renal anomalies, polydactyly, learning disabilities, and reproductive abnormalities [2]. In addition to these primary features, secondary characteristics such as cardiovascular disease, hearing loss, and dental anomalies have also been reported. The etiology of BBS is attributed to mutations in the BBS genes, of which most are crucial for the proper structure and function of cellular cilia. Mutations in these genes mainly impair ciliary function, leading to disruptions in chemical signaling pathways during development. This ciliary dysfunction underlies the broad phenotypic spectrum of BBS and complicates the diagnostic process, particularly in pediatric populations. A widely used diagnostic criteria for BBS was established through the work of Beales et al. [3]. However, the complicated and resource-intensive diagnostics often contribute to frequent delays in diagnosis, which subsequently postpones the initiation of appropriate management strategies and results in poorer patient outcomes [4]. Therefore, there is an urgent need to improve the ease of diagnosis of BBS to minimize these delays and initiate treatment.

In our clinical practice, we have observed a potential association between the presence of melanocytic nevi and BBS. This observation is supported by the work of Choi et al., which suggests a connection between ciliary function and melanogenesis, and by Haws et al., who documented cutaneous findings in patients with BBS [5, 6]. Based on these findings, we set out to assess the rates of congenital melanocytic nevi in BBS patients registered with the Clinical Registry for Bardet-Biedl Syndrome (CRIBBS) database. Incorporating a low resource assessment at birth may enhance the sensitivity of early diagnosis, potentially improving clinical outcomes for affected individuals.

## Materials and methods

### Study design and participants

The study utilized an online survey of Bardet-Biedl Syndrome (BBS) patients from the Clinical Registry Investigating Bardet-Biedl Syndrome (CRIBBS) database. Using a cross sectional design, the primary outcome of the survey was to determine the presence or absence of melanocytic nevi within this patient population. This survey included the question, “Do you have a brown or black birthmark? (Including underneath nails.)” to determine such presence. The relative size and anatomical location of the melanocytic nevi, when present, were also addressed with additional descriptors of interest.

In addition, the secondary outcome of the study involved the prevalence of major and minor symptoms of the diagnostic criteria. This outcome was used to determine if a higher prevalence of such symptoms were reported among participants who had melanocytic nevi compared to those who did not. This study was approved by the University of Oklahoma IRB.

### Survey questionnaire

Participants in CRIBBS are offered the opportunity to consent to being contacted about opportunities to participate in research studies related to BBS. The survey (see Supplemental Table 1) was emailed to the patient population who had consented to be contacted with a link to a REDCap survey and was open from 3/20/2024 to 4/4/2024. The inclusion criteria for the study was an affirmation of a self-report diagnosis of BBS in response to the question, “Have you been diagnosed with BBS?”.

Survey items assessed the following: age, gender, race, age at BBS diagnosis, if the participant had been genetically tested for BBS and if so, what gene mutations they had (if any), along with family history of BBS, and presence of BBS symptoms experienced. In addition, survey questions included information regarding melanocytic nevi (size, location, personal history of melanoma). Participants who reported a melanocytic nevi of dime size were considered small in size, while those greater than dime size were considered medium sized.

### Statistical analysis

Descriptive statistics were presented as counts and percentages for categorical variables; means and the standard deviation were presented for continuous variables. To test for an association between melanocytic nevi and categorical variables of interest, a Chi-square test or Fisher’s Exact test were used; Fisher’s Exact test was used when expected sample sizes were less than five in more than 20% of cells in the table. To test for an association between melanocytic nevi and continuous variables of interest, independent sample t-tests were utilized, as the assumption of normality was not violated in any analysis.

Odds ratios (ORs) and 95% Confidence Intervals (CI) were implemented to show the strength of associations for significant Chi-square test results, while *Cohen’s d* was applied to show the strength of association for significant t-test results. The questions over BBS symptoms were asked in a Yes/No format, and missing values for a symptom were imputed as no symptom present. All statistical analysis was conducted using R version 4.2.2, with differences considered statistically significant at *p* < 0.05.

## Results

72 initial responses were submitted. Five patients did not report a BBS diagnosis and were excluded. Among the 67 participants included in the analysis, the average age was 21 years old (*SD* = 14.5), while the majority of the sample identified as female (58.2%, *n* = 39), and Caucasian (85.1%, *n* = 57). The participants in this study were similar in age to the CRIBBS cohort as a whole (*n* = 760; mean age 23.1 years; *SD* = 14.9). Of the participants in the CRIBBS cohort, 48.9% identify as female, and 78% Caucasian. Over half the sample reported being diagnosed with BBS between 1 and 5 years of age (37.3%, *n* = 25) or 6–10 years of age (22.4%, *n* = 15). 23.9% (*n* = 16) reported having melanocytic nevi. Amongst the participants that reported having melanocytic nevi, 10.5% (*n* = 7) reported having a small sized melanocytic nevus and 13.4% (*n* = 9) reported having a medium sized melanocytic nevus. In addition, the most common anatomical locations reported for these patients were the legs (37.5%, *n* = 6) and the torso (31.3%, *n* = 5). These descriptive statistics on demographic and melanocytic nevi characteristics can be found in Table 1**.**

**Table 1:** Demographic characteristics and melanocytic nevi characteristics of the sample

Variable	% (n)
Age (yr), Mean (SD)	21.1 (14.5)
*Gender*	
Another gender	1.5 (1)
Female	58.2 (39)
Male	40.3 (27)
*Race*	
Asian	3.0 (2)
Hispanic	3.0 (2)
Indigenous	1.5 (1)
Multiracial	4.5 (3)
Other	3.0 (2)
Caucasian	85.1 (57)
*BBS age diagnosis (years)*	
< 1	9.0 (6)
1–5	37.3 (25)
6–10	22.4 (15)
11–15	6.0 (4)
16–20	11.9 (8)
21 +	13.4 (9)
Do you have a brown or black birthmark (melanocytic nevi)?	23.9 (16)
*Size of melanocytic nevi*	
Dime-sized (small)	10.5 (7)
Quarter, half-dollar, larger than half-dollar-sized (medium)	13.4 (9)
Melanocytic nevi location*	
Arms	6.3 (1)
Legs	37.5 (6)
Torso	31.3 (5)
Face	18.8 (3)
Multiple places	6.3 (1)
Personal history of melanoma	4.5 (3)

*Denominator for percentage calculation was n = 16 for percentage of those with melanocytic nevi

Almost all participants reported being genetically tested for BBS (92.5%, *n* = 62). No statistically significant association was seen between a family history of BBS and melanocytic nevi (*p* = 0.769). Among nineteen participants with a family member who had been diagnosed with BBS, 26.3% (*n* = 5) had melanocytic nevi, compared to 22.9% (*n* = 11) of the participants without a family history of BBS (*n* = 48). No statistically significant association was present between any gene mutation and melanocytic nevi, though among those with melanocytic nevi, 37.5% (*n* = 6) had BBS10 mutations compared to 27.5% (*n* = 14) among those without melanocytic nevi. The most common gene mutations amongst the entire sample (*n* = 67) were BBS1 (40.3%, *n* = 27), and BBS10 (30.0%, *n* = 20). Additionally, though not statistically significant (*p* = 0.152), there was a higher prevalence of melanocytic nevi reported among participants who identified as male (33.3%, *n* = 9) compared to those who identified as female (17.9%, *n* = 7), as seen in Supplemental Table 2. There was not a significant difference observed in average age between those with and those without melanocytic nevi (*p* = 0.520), though participants with melanocytic nevi had a higher average age (*M* = 23.2, *SD* = 13.6) compared to patients without melanocytic nevi (*M* = 20.5, *SD* = 14.8).

**Fig. 1:**
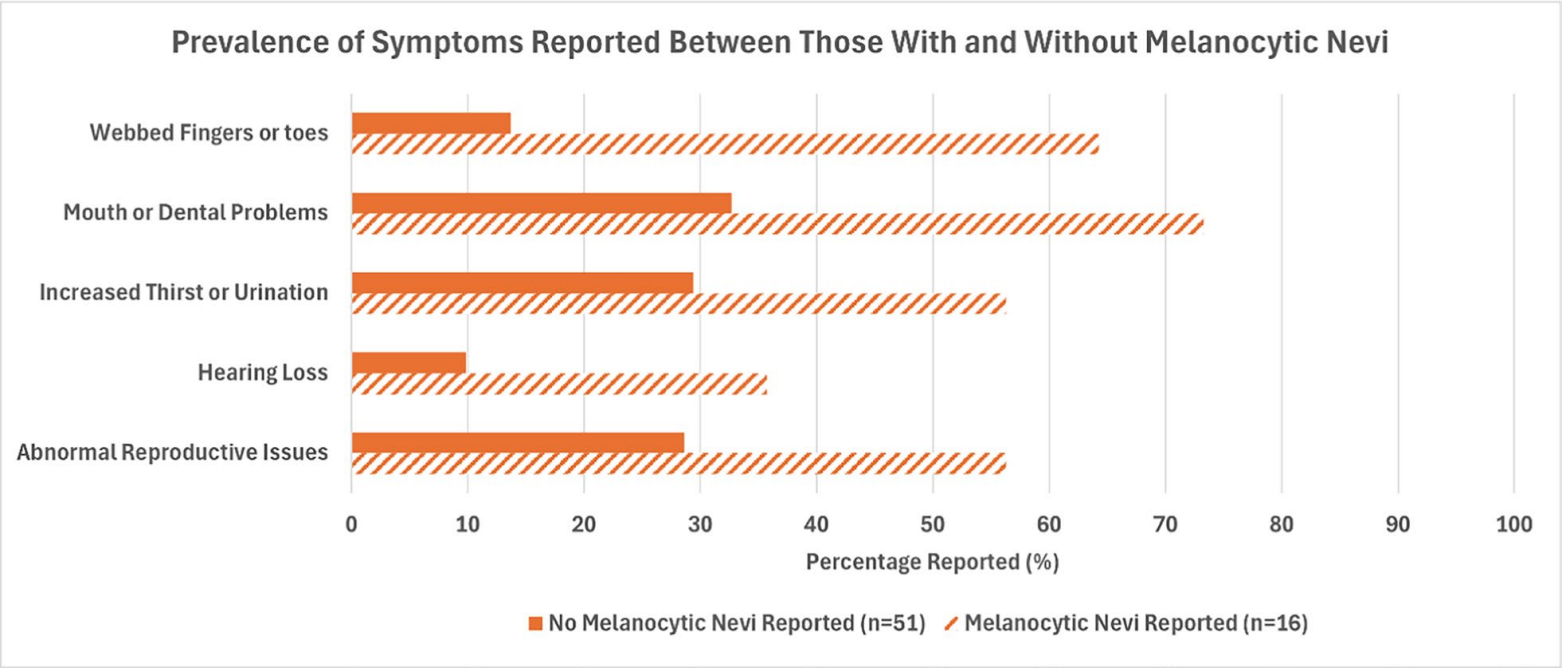
Prevalence of symptoms reported between those with and without melanocytic nevi. This indicates a higher prevalence of webbed fingers or toes, mouth or dental problems, increased thirst or urination, hearing loss, and abnormal reproductive issues was observed in survey participants with melanocytic nevi. These differences were significant other than hearing loss (*p* = 0.050) and increased thirst or urination (*p* = 0.051) evaluated by a Chi-square test or Fisher’s Exact test

When *p* < 0.10 between the BBS symptom reported and melanocytic nevi and a large enough sample size was available to use a Chi-Square test, both Odds Ratios (OR) and 95% confidence intervals were reported. Significantly higher prevalences of abnormal reproductive health issues (*p* = 0.034), high arched palate, missing teeth, dental crowding, short teeth roots (*p* = 0.008), and webbed fingers or toes (*p* = 0.001) were reported among those with melanocytic nevi compared to those without. Participants with melanocytic nevi had 3.40 (95% CI: 1.06, 10.88) times the odds of reporting abnormal reproductive health issues, and 4.81 (95% CI: 1.43, 16.16) times the odds of reporting high arched palate, missing teeth, dental crowding, or short teeth roots compared to those without melanocytic nevi. Among participants with melanocytic nevi, 56.3% (*n* = 9) reported having webbed fingers or toes, while only 13.7% (*n* = 7) reported having webbed fingers or toes among participants without melanocytic nevi. Though not statistically significant, there was a trend of a higher prevalence of hearing loss (*p* = 0.050) and increased thirst or increased urination (*p* = 0.051) reported among those with melanocytic nevi compared to those without. Comparisons for each can be seen in Table 2 and Fig. 1.

**Table 2:** Overall prevalence of BBS symptoms and association with melanocytic nevi

Variable	Overall sample % (n)	Melanocytic nevi (*n* = 16) % (n)	No melanocytic nevi (*n* = 51) % (n)	*p*-value	OR (95% CI)
*BBS symptom*					
Legal blindness	76.1 (51)	81.3 (13)	74.5 (38)	0.742*	
Obesity	85.1 (57)	93.8 (15)	82.4 (42)	0.430*	
Born w/extra fingers or toes	74.6 (50)	68.8 (11)	76.5 (39)	0.528*	
Abnormal reproductive issues	34.3 (23)	56.3 (9)	27.5 (14)	0.034	3.40 (1.06, 10.88)
Learning difficulties	77.6 (52)	87.5 (14)	74.5 (38)	0.492*	
Kidney abnormalities	37.3 (25)	50.0 (8)	33.3 (17)	0.229	
Type 2 diabetes	4.5 (3)	6.3 (1)	3.9 (2)	NA**	
Heart disease	10.5 (7)	18.8 (3)	7.8 (2)	0.345*	
Hearing loss	14.9 (10)	31.3 (5)	9.8 (5)	0.050*	
Speech deficiency	50.8 (34)	56.3 (9)	49.0 (25)	0.614	
Increased thirst or increased urination	35.8 (24)	56.3 (9)	29.4 (15)	0.051	3.09 (0.97, 9.81)
Developmental delay	71.6 (48)	75.0 (12)	70.6 (36)	1.00*	
Muscle spasms	17.9 (12)	25.0 (4)	15.7 (8)	0.460*	
Imbalance or poor coordination	70.2 (47)	75.0 (12)	68.6 (35)	0.760*	
High arched palate, missing teeth, dental crowding, short teeth roots	40.3 (27)	68.8 (11)	31.4 (16)	0.008	4.81 (1.43, 16.16)
Abnormally short fingers or toes	41.8 (28)	50.0 (8)	39.2 (20)	0.445	
Webbed fingers or toes	23.9 (16)	56.3 (9)	13.7 (7)	0.001*	
Astigmatism or cataracts or lazy eye	58.2 (39)	56.3 (9)	58.8 (30)	0.855	
Liver abnormalities	13.4 (9)	25.0 (4)	9.8 (5)	0.201*	

*Fisher’s exact test used because expected sample size < 5 in greater than 20% of cells

**Expected sample size too small in at least one cell in 2 × 2 table to use Fisher’s exact test (< 1)

**Fig. 2:**
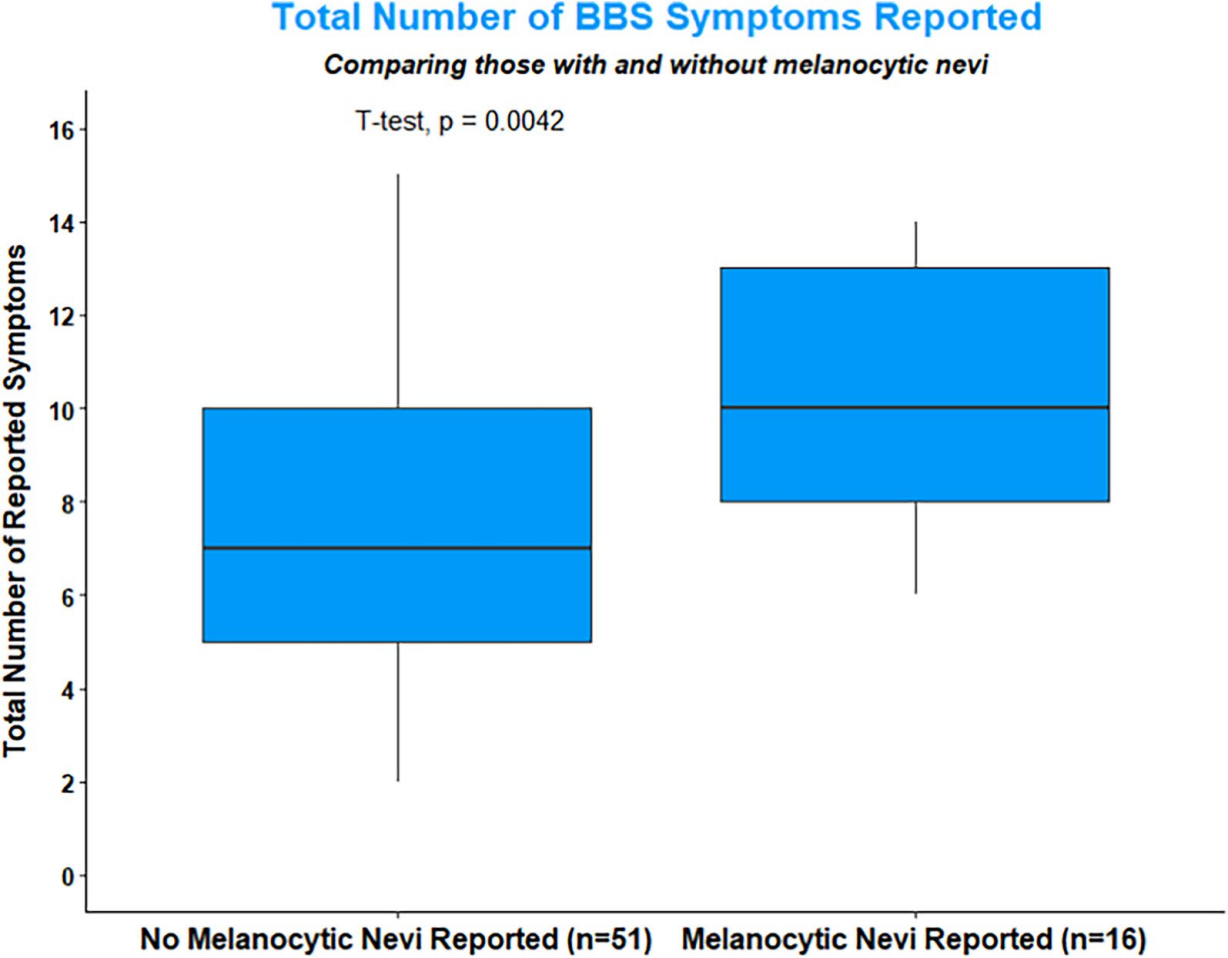
Total Number of BBS symptoms reported between those with and without melanocytic nevi. This indicates a significantly higher number of symptoms were reported in survey participants with melanocytic nevi. This difference was significant evaluated by an independent sample t-test

No significant association was present between meeting the clinical diagnostic criteria for BBS and melanocytic nevi (*p* = 0.267); however, there was a higher number of total BBS symptoms (major and minor) reported amongst individuals with melanocytic nevi (*M* = 10.4, *SD* = 2.9), compared to those without melanocytic nevi (*M* = 7.7, *SD* = 3.1), seen in Table 3.^3^ This finding was statistically significant with a large effect size (*t*(65) = 2.97, *p* = 0.004, *Cohen’s d* = 0.85)(Fig. 2).

**Table 3:** Association between number of symptoms and melanocytic nevi

Variable	Overall sample	Melanocytic nevi % (n)	No melanocytic nevi % (n)	*p*-value	OR (95% CI)|*Cohen’s d*
# of BBS symptoms (major or minor feature)—mean (SD)	8.4 (3.2)	10.4 (2.9)	7.7 (3.1)	0.004	0.85
# of BBS symptoms (major or minor feature) collapsed					
9 +	46.3 (31)	35.5 (11)	64.5 (20)	0.039	3.41 (1.03, 11.29)
0–8	53.7 (36)	13.9 (5)	86.1 (31)		
Met clinical diagnostic criteria					
Yes	82.1 (55)	27.3 (15)	72.7 (40)	0.267	
No	17.9 (12)	8.3 (1)	91.7 (11)		
# of major BBS symptoms reported—mean (SD)	3.9 (1.3)	4.4 (1.4)	3.7 (1.3)	0.068	0.53

After collapsing symptoms into categories of 0–8 versus ≥ 9, those with melanocytic nevi had 3.41 (95% CI: 1.03, 11.29) times the odds of reporting ≥ 9 BBS symptoms compared to those without melanocytic nevi. When limiting the number of symptoms to major BBS symptoms reported, there was a higher number of major symptoms reported among individuals with melanocytic nevi (*M* = 4.4, *SD* = 1.4) compared to participants without melanocytic nevi (*M* = 3.7, *SD* = 1.3), though this finding was not statistically significant (*p* = 0.068).

## Discussion

In 1999, Beales et. al. revised the diagnostic criteria for BBS in order to diagnose affected individuals earlier. These criteria, still in use today, were developed based on symptoms reported by 109 surveyed BBS patients. From this data, Beales et. al. determined that diagnosing BBS would be based on 6 major and 11 minor criteria. The major criteria, or primary features, of BBS include rod-cone dystrophy, polydactyly, obesity, learning disabilities, hypogonadism, and renal anomalies. The minor criteria, or secondary features of BBS, include speech disorder, strabismus/cataracts/astigmatism, brachydactyly/syndactyly, developmental delay, polyuria/polydipsia, ataxia/poor coordination/imbalance, limb spasticity, diabetes mellitus, dental/palate abnormalities, congenital heart disease/left ventricular hypertrophy, and hepatic fibrosis [3]. In 2024, Dollfus et al. published a new recommended diagnostic criteria for BBS taking into account genetic testing, age, primary features, and secondary features. This criteria was created from an expert consensus with less diagnostic features added to simplify the use of the criteria [7].

Our survey found that 23.9% of our 67 surveyed BBS participants reported congenital melanocytic nevi (CMN). This finding matches the data collected by Beales et. al., who found 22% of their 109 BBS patients reporting pigmented nevi [3]. In the general population, small CMN (less than 1.5 cm in diameter) are seen in 1% of newborns and medium CMN (1.5 to 19.9 cm in diameter) are seen in 0.1% of newborns [8]. In contrast, our surveyed participants reported 10.5% small CMN and 13.4% medium CMN, both reported at higher rates than that of the general population. Therefore, we believe the genetic mutations of BBS may play a role in the formation of CMN.

Our study also revealed a link between CMN and certain BBS phenotypes, as seen in Table 2**.** Our data showed that BBS patients with CMN were more likely to have reproductive abnormalities, hearing loss, palate/tooth abnormalities, and webbed fingers/toes. This link could be related to the effect that BBS proteins have on neural crest cell (NCC) migration. By using a fish model, Tobin et. al. found that BBS proteins are required for accurate NCC migration, specifically involving craniofacial structures. He discovered that a lack of BBS proteins alters non-canonical Wnt signaling essential for early NCC migration. He also described that aberrant Sonic Hedgehog (SHH) signaling leads to disruption in NCC migration and is a major contributor to craniofacial defects in BBS patients [9]. Moreover, Seo et. al. found leucine-zipper transcription factor-like protein (LZTFL1) that is responsible in downregulating BBS protein complexes, limiting its ciliary access [10]. CMN are thought to originate from abnormal migration of melanocytes in the neural crest [11]. NCCs contribute to the development of many structures of the outer ear, middle ear, and inner ear; therefore alterations in NCC migration could contribute to hearing loss [12]. Gonadotropin releasing hormone (GnRH) neurons also have an NCC origin and control reproductive function [13]. Finally, syndactyly seen in patients with BBS could be due to the effect BBS proteins have on non-canonical Wnt signaling and SHH signaling, as this signaling is required for interdigital cell death [14]. These findings justify future research into the association between certain BBS phenotypes and genotypes and CMN.

Participants with CMN in our study were more likely to have more BBS symptoms than those without CMN. We grouped BBS symptoms into two groups: those with 0–8 symptoms and those with 9 or more symptoms. The results of our survey revealed that those with CMN were more likely to have 9 or more symptoms related to BBS. This may be due to the effects BBS has on neural crest cell migration in embryologic development leading to CMN and other neural crest cell derived abnormalities. However, future research is needed on the connection between symptom burden in BBS and CMN, considering the mutated BBS gene and presence or absence of other genetic modulators.

We also found that participants with mutations in BBS10 had a higher percentage of CMN. This finding was not statistically significant; however, this is likely due to our low sample size. BBS10 is one of the more common genetic mutations found in BBS patients, therefore this increased percentage of CMN could also be attributed to more patients having that mutation. Larger trials are needed to determine if BBS10 is linked to CMN, with a focus on correlation between severe, truncating mutations and CMN. In addition, due to the small number of participants with more than one BBS gene mutation in the study, we were unable to assess the relationship between mutational load and CMN. Future, larger studies are needed to assess this link. There were also more males with CMN than females in our study population. This also did not have a statistically significant difference, likely due to our small sample size; however, future trials could explore this connection between gender and CMN with a larger survey population.

Despite the improved diagnostic criteria from Beales et. al. as well as Dollfus et al., the diagnosis of BBS remains difficult and delayed. Factors potentially delaying recognition and diagnosis of BBS include its wide spectrum of clinical features as well as the fact that some features, including speech delays and rod-cone dystrophy, are not detected until early childhood or later. Therefore, identifying BBS characteristics that are present at birth or in early infancy can reduce the delay in diagnosis. Additionally, once a diagnosis has been made determining which features are most likely is a challenge as considerable diversity in presentation occurs even within families. Since CMN present earlier than many of the organ manifestations of BBS, we believe screening for CMN in combination with other traditional congenital and developmental abnormalities may help identify patients whose developmental trajectory is concerning for BBS and should be referred for more resource intensive/invasive testing to evaluate for other clinical criteria. In addition, for patients presenting later in life, history and skin exam would allow for additional clinical information that may help determine if genetic testing is indicated. A limitation of our study is the low sample size of patients with CMN present in the survey. Because of this, our findings should be interpreted with caution until these findings are repeated with a larger sample. An additional limitation of this study is the lack of certainty in the diagnosis of CMN in these patients as we did not require a specialist to diagnose CMN. As this study gathered data via survey responses from participants, we relied on the participants themselves to state if they have a brown or black birthmark and the size of the birthmark. Consequently, to aid in a more sensitive and swift diagnosis of BBS, we propose to consider utilizing melanocytic nevi as an at-birth screening tool or for use in obesity clinics to help assess if individuals should receive further testing to evaluate for BBS.

## Conclusion

Our study found that patients with BBS have a higher rate of CMN than that seen in the general population. This could be due to the genetic mutations of BBS playing a role in CMN formation, possibly through NCC migration, though studies of a larger sample size are needed to confirm this hypothesis. Therefore, we recommend a CMN skin exam as a potential non-invasive screening tool to aid in earlier diagnosis.

## Supplementary Information

Supplementary file1.

## Data Availability

The data supporting the findings in this study are not publicly available due to the need to protect study participant privacy. De-identified data can be provided upon reasonable request to the corresponding author mailto:jesse-r-richards@ouhsc.edu?subject = BBS Nevi Data.
