# Supplementary material for: Congenital melanocytic nevi in Bardet-Biedl syndrome
Source: Orphanet J Rare Dis. 2025 Aug 28;20:462. doi: 10.1186/s13023-025-03870-6 (PMC12395923; doi:10.1186/s13023-025-03870-6)
Supplement: Supplementary file 1 — Supplementary file1. [file 13023_2025_3870_MOESM1_ESM.docx]

**Supplemental Table 1**: Survey Questions from Study Questionnaire

| Survey Question | Response Method |
| --- | --- |
| What is your age? | Text, Required |
| What is your gender? | Checkbox, Required  1. Male  2. Female  3. Another Gender |
| What is your race/ethnicity? Please check all that apply. | Checkbox, Required  1. Asian  2. Black  3. Hispanic  4. Indigenous  5. White  6. Another race/ethnicity |
| Another race/ethnicity | Text |
| Have you been diagnosed with BBS? | Yes/No, Required |
| What age did you find out you had BBS? | Text, Required |
| Were you genetically tested for BBS? | Yes/No, Required |
| What gene do you have? | Text, Required |
| Have any of your biological family members been diagnosed with BBS? | Checkbox, Required  1. Father  2. Mother  3. Sister(s)  4. Brother(s)  5. Son(s)  6. Daughter(s)  7. Granddaughter(s)  8. Granson(s)  9. Niece(s)  10. Nephew(s)  11. Half-brother(s)  12. Half-sister(s)  13. Maternal Grandmother  14. Maternal Grandfather  15. Maternal Aunt(s)  16. Maternal Uncle(s)  17. Maternal Cousin(s)  18. Paternal Grandmother  19. Paternal Grandfather  20. Paternal Aunt(s)  21. Paternal Uncle(s)  22. Paternal Cousin(s)  23. None  24. Don’t Know  25. Refused to Answer |
| Number of sisters with BBS | Text, Required |
| Number of bothers with BBS | Text, Required |
| Number of sons with BBS | Text, Required |
| Number of daughters with BBS | Text, Required |
| Number of granddaughters with BBS | Text, Required |
| Number of grandsons with BBS | Text, Required |
| Number of nieces with BBS | Text, Required |
| Number of nephews with BBS | Text, Required |
| Number of half-brothers with BBS | Text, Required |
| Number of half-sisters with BBS | Text, Required |
| Number of maternal aunts with BBS | Text, Required |
| Number of maternal uncles with BBS | Text, Required |
| Number of maternal cousins with BBS | Text, Required |
| Number of paternal aunts with BBS | Text, Required |
| Number of paternal uncles with BBS | Text, Required |
| Number of paternal cousins with BBS | Text, Required |
| Mark all BBS symptoms that you experience: |  |
| Legal blindness (including retinal degeneration and night blindness) | Yes/No |
| Obesity | Yes/No |
| Born with extra fingers or toes | Yes/No |
| Abnormal reproductive issues (including infertility, abnormally small testicles, undescended testicles; underdeveloped uterus, fallopian tubes, or ovaries) | Yes/No |
| Learning difficulties (including delayed speech or reading skills, a learning disability, or intellectual disability) | Yes/No |
| Kidney abnormalities (including kidney malformations or kidney disease) | Yes/No |
| Type 2 diabetes | Yes/No |
| Heart disease | Yes/No |
| Hearing loss | Yes/No |
| Speech deficiency | Yes/No |
| Increased thirst or increased urination | Yes/No |
| Developmental Delay | Yes/No |
| Muscle spasms | Yes/No |
| Imbalance or poor coordination | Yes/No |
| High arched palate, missing teeth, dental crowding, short teeth roots | Yes/No |
| Abnormally short fingers or toes | Yes/No |
| Webbed fingers or toes | Yes/No |
| Astigmatism or cataracts or lazy eye | Yes/No |
| Liver abnormalities (including liver malformations or liver disease) | Yes/No |
| Do you have a brown or black birthmark? (including underneath nails) | Yes/No, Required |
| Location on your body? | Text, Required |
| Size? | Checkbox, Required   1. Dime-sized 2. Quarter-sized 3. Half-dollar sized 4. Larger than the above |
| Approximate size of birthmark in centimeters: | Text, Required |
| Personal history of melanoma? | Yes/No, Required |

**Supplemental Table 2**. Prevalence of genetic testing and family history of BBS, association with genetic mutations and gender with melanocytic nevi

| Variable | Overall Sample  % (n) | Melanocytic Nevi (n=16)  % (n) | No Melanocytic Nevi (n=51)  % (n) | p-value |
| --- | --- | --- | --- | --- |
| Genetically tested for BBS |  |  |  |  |
| Yes | 92.5 (62) | - | - | - |
| No | 7.5 (5) | - | - |  |
| Any Family Member w/ a BBS diagnosis? |  |  |  |  |
| Yes | 28.4 (19) | 26.3 (5) | 73.7 (14) | 0.769 |
| No | 71.6 (48) | 22.9 (11) | 77.1 (37) |  |
| GENES Mutations (not mutually exclusive) |  |  |  |  |
| BBS1 | 40.3 (27) | 31.3 (5) | 43.1 (22) | 0.398 |
| BBS2 | 9.0 (6) | 6.3 (1) | 9.8 (5) | 1.00* |
| BBS 7 | 3.0 (2) | 12.5 (2) | 0.0 (0) | NA** |
| BBS10 | 30.0 (20) | 37.5 (6) | 27.5 (14) | 0.534 |
| Other (BBS4, BBS5, PCSK1, BBS6, BBS9, LB12, MKKS) | 9.0 (6) | 6.2 (1) | 9.8 (5) | 1.00* |
| No mutation, unknown, unsure, missing | 11.9 (8) | 12.5 (2) | 11.8 (6) | 1.00* |
| Gender |  |  |  |  |
| Female | 59.1 (39) | 17.9 (7) | 82.1 (32) | 0.152 |
| Male  Age (yr), Mean (SD) | 40.9 (27)  21.1 (14.5) | 33.3 (9)  23.2 (13.6) | 66.7 (18)  20.5 (14.8) | 0.520 |

*Fisher’s exact test used because expected sample size < 5 in greater than 20% of cells

**Expected sample size too small to use Fisher’s exact test (< 1 in at least one cell)
